# CRISPR/Cas9n-Mediated Deletion of the Snail 1Gene (*SNAI1*) Reveals Its Role in Regulating Cell Morphology, Cell-Cell Interactions, and Gene Expression in Ovarian Cancer (RMG-1) Cells

**DOI:** 10.1371/journal.pone.0132260

**Published:** 2015-07-10

**Authors:** Misako Haraguchi, Masahiro Sato, Masayuki Ozawa

**Affiliations:** 1 Department of Biochemistry and Molecular Biology, Graduate School of Medical and Dental Sciences, Kagoshima University, Kagoshima, Japan; 2 Section of Gene Expression Regulation, Frontier Science Research Center, Kagoshima University, Kagoshima, Japan; University of Colorado, Boulder, UNITED STATES

## Abstract

Snail1 is a transcription factor that induces the epithelial to mesenchymal transition (EMT). During EMT, epithelial cells lose their junctions, reorganize their cytoskeletons, and reprogram gene expression. Although Snail1 is a prominent repressor of E-cadherin transcription, its precise roles in each of the phenomena of EMT are not completely understood, particularly in cytoskeletal changes. Previous studies have employed gene knockdown systems to determine the functions of Snail1. However, incomplete protein knockdown is often associated with these systems, which may cause incorrect interpretation of the data. To more precisely evaluate the functions of Snail1, we generated a stable cell line with a targeted ablation of Snail1 (Snail1 KO) by using the CRISPR/Cas9n system. Snail1 KO cells show increased cell–cell adhesion, decreased cell–substrate adhesion and cell migration, changes to their cytoskeletal organization that include few stress fibers and abundant cortical actin, and upregulation of epithelial marker genes such as E-cadherin, occludin, and claudin-1. However, morphological changes were induced by treatment of Snail1 KO cells with TGF-beta. Other transcription factors that induce EMT were also induced by treatment with TGF-beta. The precise deletion of Snail1 by the CRISPR/Cas9n system provides clear evidence that loss of Snail1 causes changes in the actin cytoskeleton, decreases cell–substrate adhesion, and increases cell–cell adhesion. Treatment of RMG1 cells with TGF-beta suggests redundancy among the transcription factors that induce EMT.

## Introduction

The epithelial-to-mesenchymal transition (EMT) is a common process that occurs during development, wound healing and cancer metastasis. During EMT, epithelial cells lose their junctions, change their shape, reorganize their cytoskeletons, and reprogram gene expression [1]. This change in gene expression is induced by several master regulators, including the Snail1, TWIST, and zinc-finger E-box binding (ZEB) transcription factors. Their contributions to the induction of EMT depend on the cell type and the signaling pathway that initiates EMT. They often control the expression of each other and functionally cooperate at target genes [1]. EMT is regulated by signaling pathways mediated by multiple cytokines, including Wnt, Notch and transforming growth factor (TGF)-beta [2]. The TGF–beta signaling pathway has a dominant role among them [1]. Upon the induction of EMT by TGF-beta, transcription factors such as Snail1 are upregulated [3]. Snail1’s role in EMT has been extensively studied. Snail1 is a strong repressor of epithelial markers such as E-cadherin, the claudins and the occludins. On the other hand, Snail1 increases the expression of mesenchymal markers such as vimentin and fibronectin [4], [5]. We have confirmed that Snail1 regulates cell-matrix adhesion through its regulation of the expression of integrin and basement membrane proteins such as the laminins [6]. Snail1 is primarily thought to induce EMT through direct repression of E-cadherin, which leads to nuclear beta-catenin translocation and further alterations in transcription [7]. Snail1 gene expression is reported to induce changes in cell shape and movements [5]. These changes require alterations in actin organization. However, the molecular mechanisms controlling F-actin dynamics during EMT are not fully understood [1]. Recently, McGrail investigated the role of Snail1 in cytoskeletal reformation and actin dynamics. They investigated the effect of Snail1 on the expression of actin-cytoskeleton-related genes [8]. We also tried to clarify the impact of Snail1 on cell shape and actin conformation by deleting Snail1 from RMG1 cells.

The function of Snail1 has been evaluated in several types of cancer cells through the use of RNA interference (RNAi) [9]. However, knockdown of target gene expression by RNAi is incomplete and temporary [10]. To determine the precise role of Snail1 during the induction of EMT, we have employed a novel genome editing system called “clustered Regularly Interspaced Short Palindromic Repeats (CRISPR)/ CRISPR-associated 9 (Cas9)” The CRISPR/Cas9 system consists of two components: the Cas9 protein and a guide RNA (gRNA). The Cas9 protein possesses nuclease activity and can induce double-stranded breaks (DSBs) in any genomic DNA sequence guided by a gRNA, provided that a protospacer adjacent motif (PAM) sequence exists at the target locus [11]. In the absence of a repair template, are ligated through a non-homologous end-joining process, which causes small insertion or deletion mutations known as indels. Thus, CRISPR/Cas9 allows for specific genomic disruption [12]. Recent studies have shown that CRISPR/Cas9 can be highly active in human cells, even with imperfectly matched RNA-DNA interfaces. Therefore, the CRISPR/Cas9 system can induce high-frequency off-target mutagenesis in human cells [13]. To avoid off-target mutagenesis, Ran et al [14] developed a strategy that combines the aspartae-to alanine (D10A) mutant nickase version of Cas9 (Cas9n) with a pair of offset gRNAs complementary to opposite strands of the target site. Cas9n nicks DNA to yield single-stranded breaks. These breaks are preferentially repaired through homology-directed repair, which decreases the frequency of unwanted indel mutations resulting from off-target DSBs [12]. When Cas9n is combined with a pair of gRNAs, double nicking causes DSBs at only the genomic loci where both of the gRNAs bind at an appropriate distance. This effectively increases the specificity of target recognition. This double nicking system is reported to reduce off-target activity by 50- to 1500-fold in cell lines [14].

In this study, we employed the CRISPR/Cas9n system to specifically block Snail1 expression in human ovarian adenocarcinoma (RMG-1) cells [15] and determined the effect of this Snail knockout (KO) on cell morphology and function, as well as TGF-beta-induced EMT.

## Materials and Methods

### Ethics statement

Experiments with recombinant DNA technology were performed in accordance with the guidelines of the Kagoshima University Committee on recombinant DNA. The security approval number is 26005.

### Cell line and culture

Human ovarian mesonephroid adenocarcinoma cell line RMG1 [15] was obtained from the JCRB (Japanese Collection of Research Bioresources, Osaka) Cell Bank and cultured in Dulbecco's Modified Eagle Medium (DMEM) (Nissui, Tokyo) supplemented with 10% fetal calf serum (FCS) (Nichirei Biosciences, Inc. Tokyo).

### Antibodies and reagents

Mouse monoclonal antibodies (mAbs) against human E-cadherin (clone 36, BD Biosciences, San Jose, CA), human Snail1 (clone L7042, Cell Signaling, Danvers, MA), human vimentin (clone V9, ZYMED, Carlsbad, CA), human vinculin (clone hVIN-1, Sigma, St. Louis, MO), human vitronectin (clone 342603, R&D systems, Minneapolis, MN), human fibronectin (clone 10/Fibronectin, BD Biosciences, San Jose, CA), as well as polyclonal antibodies (pABs) against human claudin-1 (clone MH25, Invitrogen, Waltham, MA), human occludin (clone Z-T22, ZYMED, Carlsbad, CA), human integrin alpha 5 (Ag0860, catalog number 0569-1-AP, Proteintech, Chicago, IL), beta-actin (catalog number GTX109639, Genetex, Irvine, CA), and alpha tubulin 4a (catalog number GTX112141, Genetex, Irvine, CA) were purchased as indicated. Anti-mouse and anti-rabbit IgG horseradish peroxidase conjugates (Jackson Immunoresearch Laborattories, Inc, West Grove, PA), rhodamine X-conjugated phalloidin (Wako, Osaka) and thiazolyl blue tetrazolium bromide (MTT, Sigma-Aldrich, St. Louis, MO) were also purchased as indicated.

### CRISPR/Cas9 plasmid design

**Fig 1:**
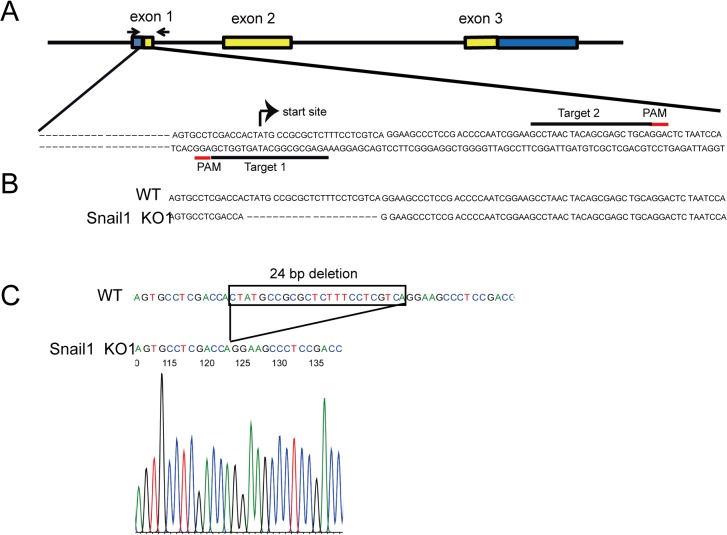
Generation of Snail1 KO cells using the CRISPR/Cas9n double nicking system. (A) Schematic illustration of Snail1 gene structure and sequences around the target loci. Yellow boxes indicate exons encoding the Snail1 protein. Blue boxes indicate non-coding exons. The gRNA target sequences and PAM domains are indicated by black and red underlining, respectively. Arrows indicate the locations of PCR primers. (B) The genomic sequences around the target sites of wild type (WT) and Snail1 KO1 cells. (C) Waveform data from a DNA sequencer displaying the sequence obtained from PCR fragments of genomic DNA.

To select the target sequence for genome editing, the human Snail1 genomic sequence around the first exon (NC_000020.11) was submitted to an online CRISPR Design Tool (http://tools.genome-engineering.org). Two target sites were selected (Fig 1A) using rules described previously [14]. The oligonucleotides used to construct gRNAs for the human Snail1 gene were: g-snail1 1s (5’- caccgAGAGCGCGGCATAGTGGTCG-3’), g-snail1 1r (5’- aaacCGACCACTATGCCGCGCTCTc-3’), g-snail1 2s (5’-caccgGCCTAACTACAGCGAGCTGC-3’) and g snail1 2r (5’- aaacGCAGCTCGCTGTAGTTAGGCc-3’).

The human codon-optimized SpCas9 nickase and chimeric guide RNA expression vector pX335-U6-Chimeric_BB-CBh-hSpCas9n (D10A), (no 42335, Addgene, Cambridge, MA) [16] was digested with *Bbs*I, (FD1014, Fermentas/Thermo Scientific, Waltham, MA) and then a pair of annealed oligonucleotides for each target site was cloned into the guide RNA according to a previously described protocol [12]. These constructs were designated pX335-Cas9-g snail1 1 and pX335-Cas9-g snail1 2, respectively.

### CRISPR/Cas9n-mediated engineering of the RMG1 cell genome

RMG1 cells (2 ×10^5^) were co-transfected with 3 μg of pX335-Cas9-g snail 1, 3 μg of pX335-Cas9-g snail 2, and 1 μg of pEGFP-N1 (Clontech, Mountain View, CA) using lipofectamine 2000 (Life Technologies, Carlsbad, CA). Two days after transfection, the transfected cells were passaged and seeded as single colonies (0.5 cells /well) in 96-well plates. On the next day, G418 (final concentration 1 mg/mL) was applied to each well. After 3–4 weeks of selection, the emerging colonies were transferred to 24 well plates to expand. After 2–3 weeks of expansion, the cells in each colony were divided in half; one half was subjected to propagation and the other half was used to assess the expression of Snail1 using western blotting. Five clones showed a significant reduction in Snail1 expression. Therefore, the genomic DNAs of these clones were isolated. DNA fragments (182 bp in size) that covered the gRNA target regions were amplified using PCR. Primers -57F (5’-GAGTGGTTCTTCTGCGCTAC-3’) and 125R (5’- CCCACGCAGCCTTCGCCTGT-3’) were used for PCR. PCR products were verified using gel electrophoresis, purified using the QIAquick gel extraction kit (Quiagen, Hilden Germany), and directly sequenced to detect the deletion mutation. PCR products from 5 clones contained deletion mutations. To confirm whether these colonies have mutations in both alleles (so-called bi-allelic KO), these PCR products were sub-cloned into the pGEM-T Easy vector (Promega Fitchburg, WI) and amplified in a bacterial host; then the plasmid DNA from individual colonies was sequenced.

### Transfection of Snail1 KO cells with Snail1 (rescue experiment)

Snail1 KO cells (2 ×10^5^) were transfected with 2.5 μg of HA-tagged human Snail1 plasmid (pCAGGS-Snail1 HA) using Lipofectamine 2000 or polyethyleneimine max (polysciences Inc, Warrington, PA). We were able to transiently transfect Snail1 KO cells with Snail1, however, we were unable to stably transfect Snail1 KO cells with Snail1.

### Western blotting

Western blotting was performed as previously described, with minor modifications [6]. Cells were boiled for 5 min in sodium dodecyl sulfate (SDS) gel sample buffer. Identical amounts of protein were separated by 10% polyacrylamide gel electrophoresis, and were transferred onto nitrocellulose membranes (Whatman Protran BA83, 0.2 μm, GE healthcare, Wauwatosa WI). Membranes were blocked with 5% non-fat milk in Tris-buffered saline (TBS), and were then incubated with specific primary antibodies (1:1000) overnight. This was followed by treatment with peroxidase-conjugated secondary antibodies. After washing with TBS, protein bands were visualized by enhanced chemiluminescence (ECL) (PRN 2106, GE Healthcare, Wauwatosa WI).

### Immunofluorescence staining

Cells were grown on coverslips for 48 h and fixed with 3% paraformaldehyde in phosphate-buffered saline (PBS) (-) for 30 min at room temperature. The cytoskeleton was visualized by staining with rhodamine X-conjugated phalloidine in PBS (-) containing 0.1% TritonX-100 for 30 min at room temperature. Cells were analyzed using a confocal laser scanning microscope (Carl Zeiss LSM700).

### RNA isolation and real-time PCR analysis

Total RNA was extracted using ISOGEN II (Wako, Osaka) and reverse-transcribed using ReverTra Ace reverse transcriptase (Toyobo, Osaka). The resulting cDNA was subjected to quantitative RT- PCR (qRT-PCR) using Syber premix Ex Taq (PR820, Takara Bio, Ohtsu). qRT-PCR was performed using the Step One Plus Real-Time PCR system (Applied Biosystems, Fostercity, CA). Expression levels were quantified using the ΔΔCt method with *GAPDH* as the calibrator. The primers used in this analysis are listed in Table 1.

**Table 1:** Primers for qRT-PCR.

*Gene name*	Forward primer	Reverse primer
*GAPDH*	5’- GCATCCTGGGCTACACTG-3’	5’- GTGAGGAGGGGAGATTCAG-3’
*snail1*	5’- ATGCCGCGCTCTTTCCTCGT-3’	5’- GCCTTTCCCACTGTCCTCATC-3’
*E-cadherin*	5’- GACACCCGATTCAAAGTGGG-3’	5’- GTCTCTCTTCTGTCTTCTGAG-3’
*claudin-1*	5’- TTGACTCCTTGCTGAATCTG-3’	5’- TTCTATTGCCATACCATGCT-3’
*occuludin*	5’- GGATCCTGTCTATGCTCATT-3’	5’- ACTGGTAACAAAGATCACCA-3’
*integrin alpha 5*	5’- ATTCTCAGTGGAGTTTTACC-3’	5’- AGAATTCGGGTGAAGTTATC-3
*beta-actin*	5’- CAAAGACCTGTACGCCAACAC-3’	5’- CATACTCCTGCTTGCTGATCC-3’
*alpha-tubulin*	5’- ATTGGCAATGCCTGCTGGGA-3’	5’- GGAAGAGCTGGCGGTAGGTG-3’
*vitronectin*	5’- CAACGTGGACAAGAAGTGCC-3’	5’- TGTCTGCTCAGGATTCCCTT-3’
*fibronectin*	5’- GTGCCTGGGCAACGGA-3’	5’- CCCGACCCTGACCGAAG-3’
*vimentin*	5’- AATGGCTCGTCACCTTCGTGAA-3’	5’- CAGATTAGTTTCCCTCAGGTTCA-3’
*ZEB1*	5’- GGTCCTCTTCAGGTGCCTCA-3’	5’- ACCAATTGAACTGATGGAGT-3’
*ZEB2*	5’- TGAGGATGACGGTATTGC-3’	5’- ATCTCGTTGTTGTGCCAG-3’
*TWIST1*	5’- GTCCGCAGTCTTACGAGGAG-3’	5’- TGGAGGACCTGGTAGAGGAA-3’
*Slug*	5’- CAAACTACAGCGAACTGGAC-3’	5’- TGAGGAGTATCCGGAAAGAG-3’
*vinculin*	5’- CCGGTGGCACAGCAGATCTC-3’	5’- AGAGGATGCCCCTTGACCCA-3’

### Attachment assay

The attachment assay was performed as previously described, with minor modifications [6]. Cells were washed and incubated with 0.125% trypsin-0.01% EDTA for 10 min at room temperature. The detached cells were collected and suspended in DMEM supplemented with 10% FCS. Cells (1.5 × 10^4^) were seeded into non-coated 96-well plates or 96-well plates that had been pre-incubated with DMEM containing 10% FCS. The same number of cells was seeded into 96-well plates that were pre-coated with poly-lysine for normalization. After 4 h incubation, non-adherent cells were removed by washing and adherent cells were fixed with 70% ethanol for 20 min. After staining with a 0.1% solution of crystal violet in PBS for 30 min, the cells were rinsed and then dissolved using 0.5% TritonX-100 in PBS. The optical density of the dissolved cells was measured at 595 nm. The number of attached cell was calculated by using a standard curve (cell number versus absorbance values). The ratio of attached cells was expressed as the number of attached cells in non-coated or pre-incubated wells divided by those in poly-lysine coated wells.

### Scattering assay

The scattering assay was performed as previously described, with minor modifications [17]. Cells were washed and incubated with 0.125% trypsin-0.01% EDTA for 10 min at room temperature. The detached cells were collected and suspended in DMEM supplemented 10% FCS. Cells (1.5 × 10^4^) were seeded into 96-well plates. After 72 h incubation, cell scattering was examined using phase-contrast microscopy. Cell scattering was quantified by counting the number of isolated cells from colonies in randomly selected phase-contrast images. The relative number of scattering was assessed by dividing the number of scattering cells derived from Snail1 KO1 or from Snail1 KO1 + Snail1 cells by the number of scattering cells derived from wild-type (WT) cells.

### Dissociation assay

The dissociation assay was performed as previously described, with minor modifications [18]. Cells (5 × 10^4^) were seeded into 24-well plates and cultured in DMEM with or without TGF-beta. After reaching confluence, the cells were washed and incubated with 0.125% trypsin or 0.125% trypsin-0.01% EDTA for 10 min at 37°C. Detached cells were carefully washed with PBS (–) and subjected to mechanical stress by pipetting with a 1 mL pipette five times. Cell fragments were fixed with 3% paraformaldehyde for 20 min and stained with crystal violet (Sigma Aldrich, St Louis MO). After careful washing, the number of particles per field was counted (minimum of five different fields). The relative number of particles is represented by the number of fragmented cell particles derived from the specified cell type divided by the number of fragmented cell particles derived from WT cells cultured without TGF-beta.

### Migration assay

The migration assay was performed as previously described, with minor modifications [6]. Migration assays were performed in Transwell chambers with an 8μm pore size (Corning, 3422, Tewksbury MA). Briefly, 0.1 ml of cells (4 × 10^5^ cells/ml) in DMEM supplemented with 0.1% bovine serum albumin (BSA) were seeded into the upper wells. In the lower wells, 0.6 ml of DMEM supplemented with 0.1% BSA containing 2.5% FCS was added. After 4 h of incubation, the non-migratory cells on the upper surface of the membrane were completely scrubbed off and 3-(4,5-dimethylthiazol-2-yl)-2,5-diphenyltetrazolium bromide (MTT) (final concentration 0.5 mg/ml) was added into the lower wells and incubated for 3 h. The migratory cells attached to the bottom surface of the membrane formed blue spots containing formazan. Those spots were photographed and counted. The relative number of migrated cells is represented by the number of migrated cells of the specified cell type divided by the number of migrated WT cells cultured without TGF-beta.

### Statistical analyses

Statistical analyses of independent samples were performed using Student’s t-test. Data are expressed as the mean ± s.e.m. Two levels of statistical significance are indicated by asterisks (**P < 0.05 and *P < 0.01).

## Results

### Generation of Snail1 KO cells using the CRISPR/Cas9n system

**Fig 2:**
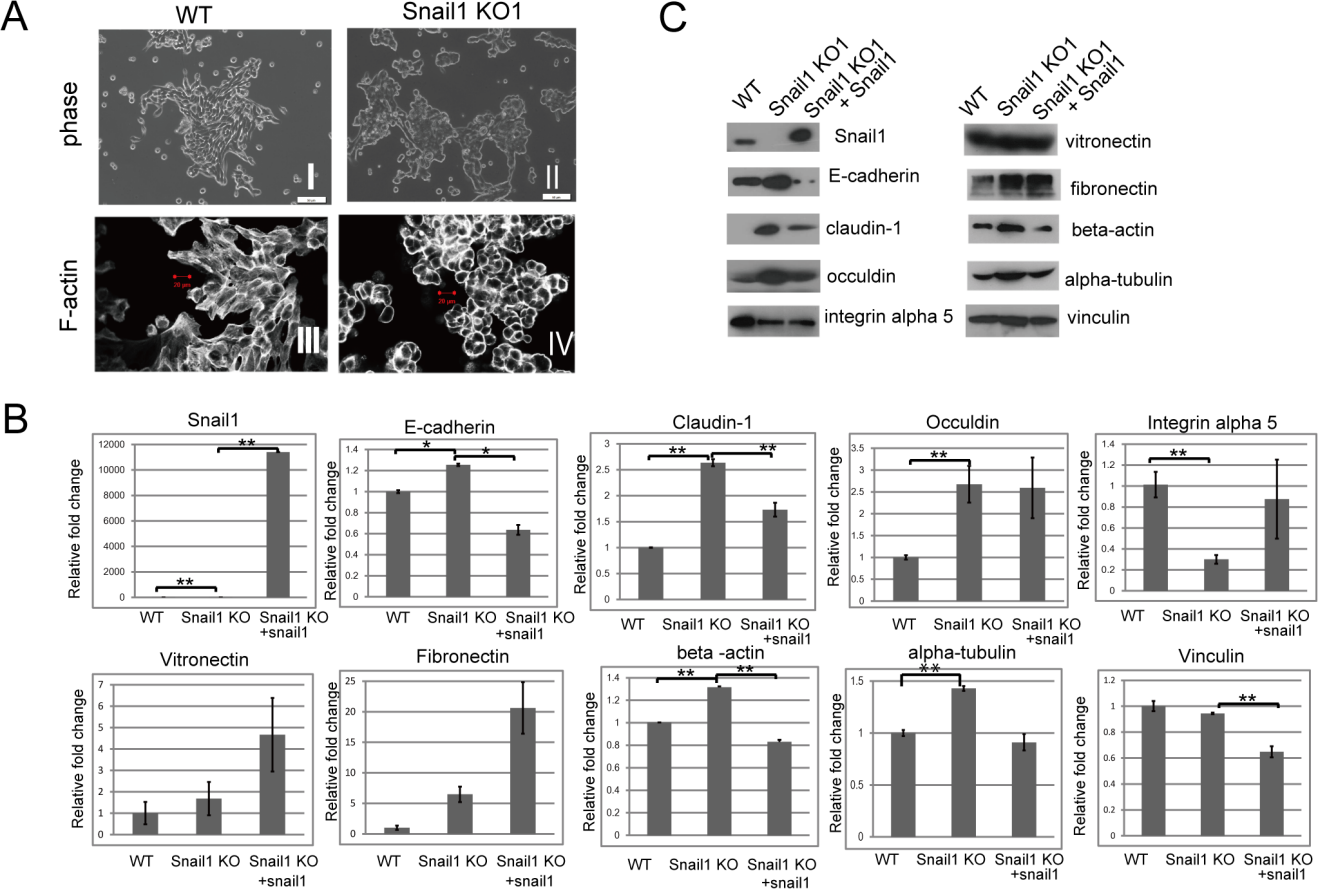
Snail1 loss altered cytoskeletal organization and gene expressions in RMG1 cells. (A) Representative images displaying the cell morphology of wild-type (WT) RMG cells (I), Snail1 KO1 cells (II). Cells are visualized by phase-contrast microscopy. Scale bar indicates 50 μm. WT cells exhibited cobblestone-like morphologies, whereas most of the Snail1 KO1 cells exhibit a more rounded morphology. Representative images of the actin cytoskeletons of WT cells (III) Snail1 KO1 cells (IV) stained with rhodamine X-conjugated phalloidine and visualized using a confocal laser scanning microscope (Zeiss LSM700). Photos of 5 to 10 images per sample were taken. Experiments were repeated 5 times. WT cells are rich in stress fibers, whereas Snail1 KO1 cells have scarce stress fibers, but cortical actin on their cell surfaces. Scale bar indicates 20 μm. (B) Quantitative RT-PCR of indicated genes in wild-type (WT) RMG cells, Snail1 KO1 cells, and Snail1 KO1 cells transiently transfected with a Snail1 expression vector (Snail1 KO1 + Snail1 cells). Values are expressed as the mean ± s.e.m of triplicate samples. Experiments were repeated three times. Statistical significance is indicated by an asterisk (*P < 0.05 and ** *P* < 0.01 using Students’*t*-test). Snail1 KO1 cells showed a complete loss of Snail expression and increased expression of *E-cadherin*, *claudin-1*, *occludin*, *beta-actin*, and *alpha-tubulin*, as well as decreased expression of *integrin alpha 5*. Transient expression of Snail1 in Snail1 KO cells reversed these effects, except for the increase in *occludin* expression. (C) Western blot analysis of indicated proteins in wild-type (WT) RMG cells, Snail1 KO1 cells, and Snail1 KO1 cells transiently transfected with a Snail1 expression vector (Snail1 KO1 + Snail1 cells). Snail1 KO1 cells showed a complete loss of Snail1 expression and an increase in E-cadherin, occludin and claudin-1, beta-actin, alpha- tubulin, as well as a decreased in integrin alpha 5 expression. Transient expression of Snail1 in Snail1 KO cells reversed these effects, except for the decreased in integrin alpha 5 expression. Vinculin was used as a loading control. Experiments were repeated three times.

To determine the roles of Snail1 during the induction of EMT, we used the CRISPR/Cas9n system to generate stable RMG1 cell lines exhibiting ablation of the gene encoding Snail1. RMG1 cells were co-transfected with pX335-Cas9-g snail1 1, which carried target 1 sequence, pX335-Cas9-g snail1 2, which carried target 2 sequence, (Fig 1A) and pEGF-N1, which carried a G418-resistance marker. Approximately 50 colonies were analyzed after G418 selection. Half of the cells in each colony were lysed, and then the lysate was used to assess Snail1 expression by western blotting. Five clones showed a significant reduction in snail expression. Therefore, genomic DNA was isolated from each of these clones. DNA fragments covering both gRNA target regions were amplified and the resulting PCR products were subjected to direct sequencing. Each of these PCR products had deletion mutations. To confirm whether both alleles had deletion mutations, the PCR products were cloned into a pGEM-T vector and amplified in a bacterial host. Plasmid DNA isolated from multiple colonies arising from each PCR product was sequenced. Two clones showed 24 bp deletions in both alleles (Fig 1B and 1C). We designated these clones Snail1 KO1 and Snail1 KO2. Each of the remaining three clones contained a WT allele. Because Snail1 KO 1 and Snail1 KO2 had the same deletion, we used Snail1 KO 1 cells in the following experiments. The absence of Snail1 expression in Snail1 KO 1 cells was confirmed by quantitative real-time PCR (Fig 2B) and western blotting (Fig 2C).

### Ablation of the Snail1 gene alters morphology and gene expression in RMG1 cells

Wild-type RMG1 cells exhibited cobblestone-like morphologies (Fig 2A–2I, S1 Fig-I), whereas most of the Snail1 KO cells exhibited a more rounded morphology (Fig 2A-II, S1 Fig-II). Because cytoskeletal organization is known to be directly linked with cell morphology, we examined the localization of the actin cytoskeleton using rhodamine X-conjugated phalloidin. WT cells showed actin stress fibers in their cytoplasms (Fig 2A-III, S1 Fig-III). In contrast, the cortical actin of Snail1 KO cells showed a thick, rounded shape (Fig 2A-IV, S1 Fig-IV). Snail1 represses the expression of epithelial markers such as E-cadherin, claudin-1 and occludin [19]. Analysis using qRT-PCR revealed that Snail1 KO1 cells exhibited increased expression of *E-cadherin*, *claudin-1*, *and occludin*. To perform a rescue experiment, a Snail1 expression vector was introduced into the Snail1 KO1 cells. Because we could not get a transfectant that exhibited stable expression of Snail1, we used transient transfectants of Snail1 (Sanil1 KO1 + Snail1) as the source of rescued Snail KO1 cells. Snail1 KO 1 + Snail1 cells showed reduced expression of *E-cadherin and claudin-1* (Fig 2B) Western blotting experiments also supported these findings. The expression of E-cadherin, claudin-1 and occludin was enhanced in the absence of Snail1 expression. Transiently transfected Snail1 in Snail1 KO1 cells supported reduced expression of E-cadherin and claudin-1 and occludin (Fig 2C). Conversely, the expression of integrin alpha 5 was reduced in Snail1 KO cells (Fig 2B and 2C). It is notable that the expression of beta-actin was affected by the presence or absence of Snail1 protein (Fig 2B and 2C).

### Ablation of the Snail gene causes alteration in cell–substratum adhesion, cell scattering, cell dissociation, and cell migration

**Fig 3:**
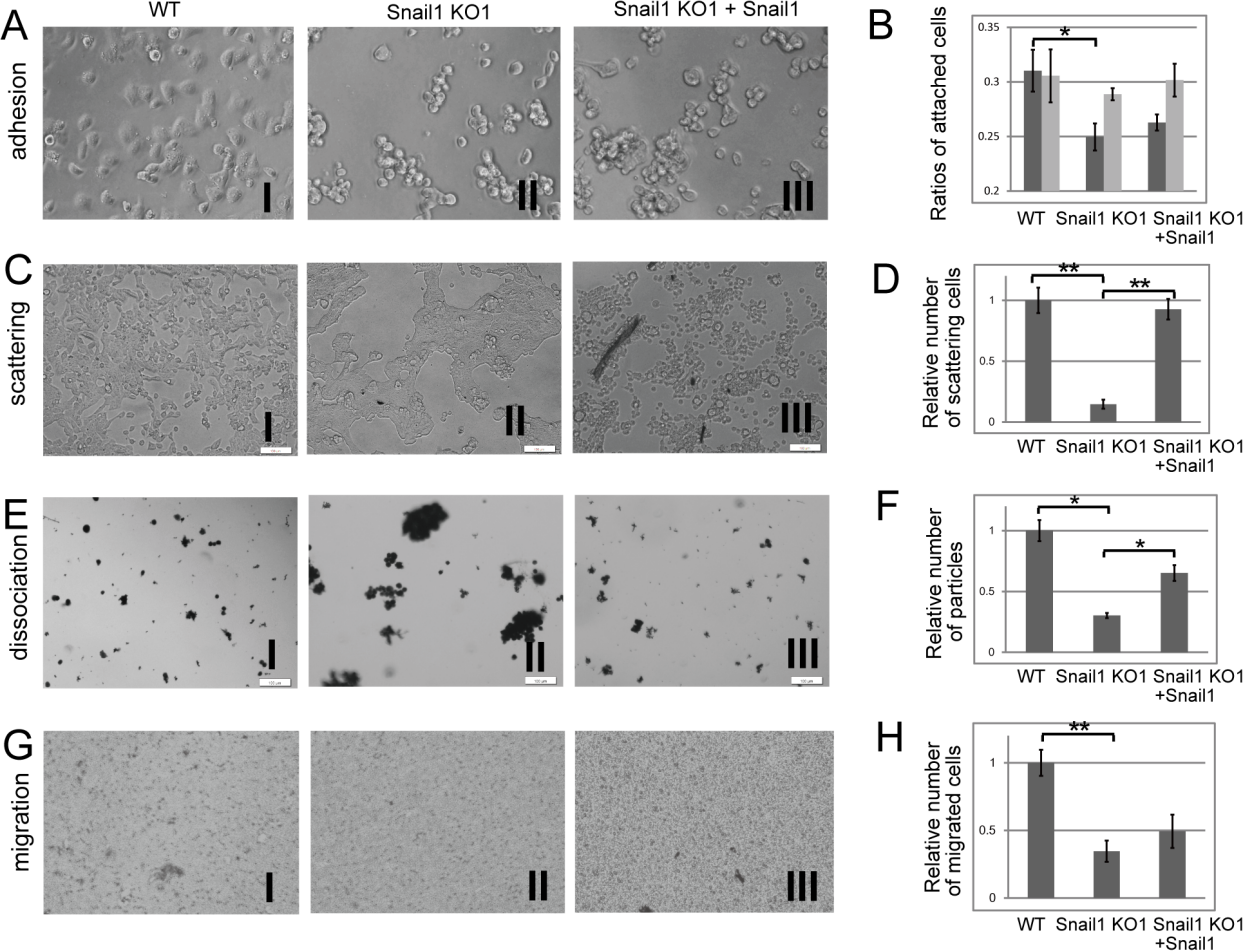
Loss of Snail1 reduced cell–substrate adhesion, cell scattering, cell dissociation, and cell migration in RMG1 cells. (A) Representative images of substrate adhesion by wild-type (WT) RMG1 cells (I), Snail1 KO1 cells (II) and Snail1 KO cells transiently transfected with a Snail1 expression vector (Snail1 KO1 + Snail1 cells) (III) 4h after seeding in non-coated wells. (B) Determination of the ratios of attached cells represented by the number of attaching cells to non-coated wells (black column) or on wells precoated with DMEM containing FCS (gray column) divided by the number attaching to wells precoated with poly-lysine. Values are expressed as the mean ± s.e.m of triplicate samples. Experiments were repeated three times. (C) Representative images of scattering cells from WT (I), Snail1 KO1 (II) and Snail1 KO1 + Snail1 (III) cells 72 h after seeding. (D) Determination of the relative number of scattering cells represented by the number of isolated cells from the colonies of each type of cells divided by the number of isolated cells from the colonies of WT cells. Values are expressed as the mean ± s.e.m of five images per sample. Experiments were repeated four times. (E) Representative images of cell dissociation among WT cells (I), Snail1 KO1 cells (II) and Snail1 KO1 + Snail1 (III) cells. (F) Determination of the relative number of fragmented particles, presented as the number of fragmented particles of each type of cells divided by the number of fragmented particles of WT cells. Values are expressed as the mean ± s.e.m of 5–10 images per sample. Experiments were repeated twice. (G) Representative images of WT cells (I), Snail1 KO1 cells (II) and Snail1 KO + Snail1 (III) cells that migrated to the lower surfaces of transwell membranes. (H) Determination of the relative number of migrated cells, presented as the number of migrated cells of each type of cells divided by the number of migrated WT cells. Values are expressed as the mean ± s.e.m. of five images per sample. Experiments were repeated three times. Statistical significance is indicated with an asterisk (*P < 0.05 and ** *P* < 0.01 using Students’ *t*-test).

Because reduced expression of integrin alpha 5 was observed in Snail1 KO1 cells, we assumed that cell–substrate adhesion might be impaired in those cells. Indeed, Snail1 KO1 cells showed significantly reduced cell–substratum adhesion compared to WT cells (Fig 3A and 3B black column). Thus, the deletion of Snail1 in RMG1 cells reduced cell–substrate adhesion. There is another possibility; that the reduction of cell–substrate adhesion in snail1 KO1 cells might result from reduction of ECM deposition. Therefore, we preincubated the wells with serum-containing medium to allow vitonectin/fibronectin to adsorb to the wells, and reexamined cell-substratum adhesion. This treatment almost rescued the reduction of cell-substratum adhesion seen with Snail1 KO1 cells. Approximately 30.6% of WT cells and 28.9% of Snail1 KO1 cells became attached to wells preincubated with serum-containing medium (Fig 3B gray column). Therefore, we investigated the expression of vitronectin and fibronectin. Contrary to expectation, Snail1 KO1 cells did not show reduced expression of these proteins (Fig 2B and 2C).

Snail1 has been reported to play a role in cell scattering [20], so we examined the scattering activity of Snail1 KO1 cells. Indeed, 72 h after seeding, WT cells displayed numerous scattering colonies (Fig 3C-I and 3D). In contrast, Snail1 KO1 cells showed a significantly lower number of scattering colonies (Fig 3C-II and 3D). Snail1 KO1 cells transfected with Snail1 recovered the scattering activity (Fig 3C-III and 3D). Thus, the deletion of Snail1 in RMG1 cells reduced cell scattering activity.

Snail1 regulates cell adhesion [5], so we assumed that cell–cell adhesion might be enhanced in Snail1 KO1 cells. To test this possibility, we performed a dissociation assay to examine the epithelial sheet integrity of Snail1 KO1 cells. Treatment with trypsin-EDTA allowed the WT cells to detach from dishes as small particles of cells. These particles dissociated completely after pipetting (Fig 3E-I and 3F). In contrast, Snail1 KO1 cells became detached in large sheets that were resistant to pipetting-based mechanical dissociation (Fig 3E-II and 3F) Transfection of Snail1 KO1 cells with Snail1 led to partial recovery of the dissociation activity (Fig 3E-III and 3F). Thus, the deletion of Snail1 in RMG1 cells increased cell–cell adhesion.

Snail1 enhances cell migration [6], so we used a transwell assay to assess the migration of Snail1 KO1 cells. Compared with WT cells, the migration of Snail1 KO1 cells towards FCS was significantly suppressed. Transfection of Snail1 KO1 cells with Snail1 led to partial recovery of the migration activity (Fig 3G and 3H). Thus, the deletion of Snail1 in RMG1 cells reduced cell migration activity.

### Loss of Snail1 did not impede TGF-beta-induced alteration of morphology and gene expression

**Fig 4:**
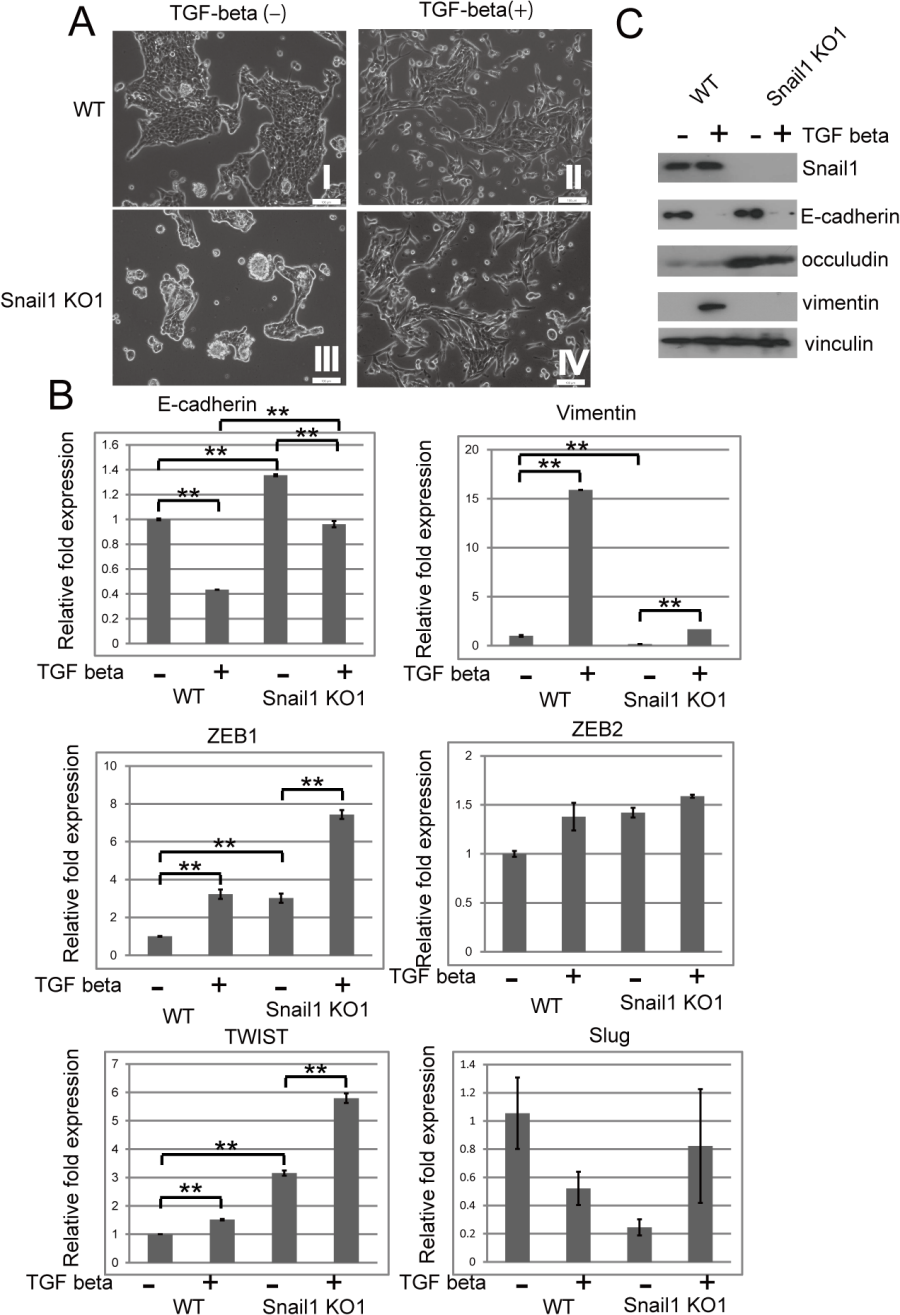
Loss of Snail1 failed to impede TGF-beta induced alterations of morphology and gene expression (A) Representative images of cell morphology of wild-type (WT) RMG (I, II) and Snail1 KO1 (III, IV) cells, after incubation for 72 h in the absence (I, III) or presence (II, IV) of TGF-beta (10 ng/mL). Scale bar indicates 100 μm. Photos were taken of 5–10 images per sample. Experiments were repeated three times. (B) Quantitative RT-PCR assessment of the cells shown in (A) for the indicated genes. Values are expressed as the mean ± s.e.m. of triplicate samples. Experiments were repeated twice. Statistical significance is indicated by an asterisk (*P < 0.05 and ** *P* < 0.01 using Students’ *t*-test). (C) Western blot analysis of the cells shown in (A) for the indicated proteins. Vinculin was used as a loading control. Experiments were repeated twice.

TGF-beta induces the expression of Snail1 [3] and Snail controls TGF-beta responsiveness [21]. Therefore, we assumed that the TGF-beta-induced EMT may be impeded in the absence of Snail1 expression. In fact, as was shown in TGF-beta treated WT cells (Fig 4A-I, II), treatment of Snail1 KO1 cells with TGF-beta induced morphological changes (Fig 4A-III, IV). TGF-beta treatment also resulted in decreased expression of E-cadherin in Snail1 KO1 cells (Fig 4B and 4C). Expression of other EMT-related transcription factors, such as *ZEB1 and TWIST*, increased in Snail1 KO1 cells, compared with WT cells, and was further enhanced by TGF-beta treatment (Fig 4B). These factors might contribute to the observed morphological changes and alterations of gene expression.

### Loss of Snail1 impedes TGF-beta-induced cell migration and cell dissociation in Snail1 KO cells

**Fig 5:**
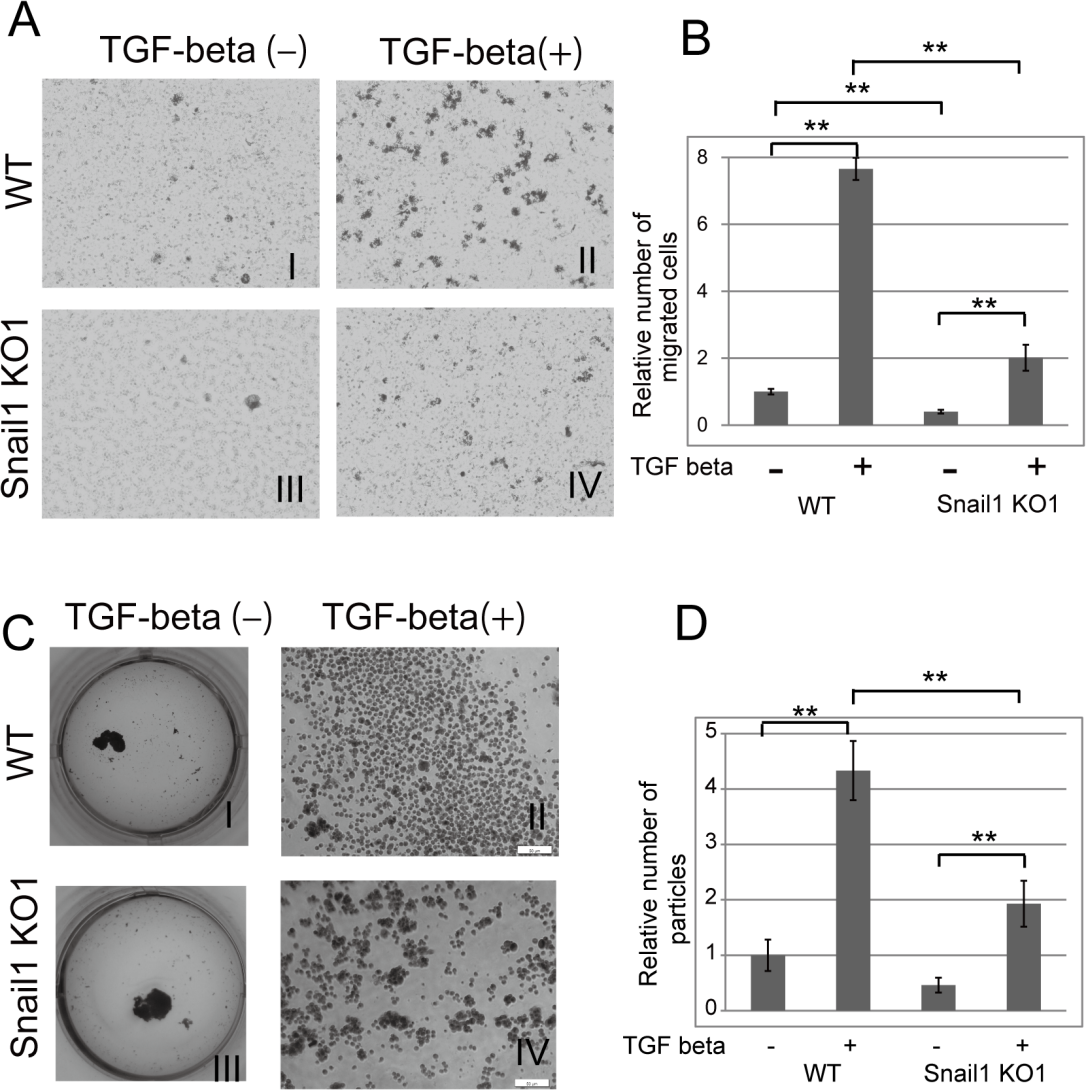
Loss of snail1 impeded TGF-beta induced cell migration and cell dissociation. (A) Representative images of wild-type (WT) RMG (I, II) and Snail1 KO1 (III, IV) cells that migrated to the lower surface of the membranes of transwell plates. Before seeding into the transwell plates, cells were incubated for 72 h in the absence (I, III) or presence (II, IV) of TGF-beta (10 ng / mL). Photos were taken of 10 images per sample. Experiments were repeated twice. (B) Determination of the relative number of migrated cells, presented as the number of migrated cells shown in (A) divided by the number of migrated WT cells incubated in the absence of TGF-beta. Values are expressed as the mean ± s.e.m. of ten images per sample. Statistical significance is indicated with an asterisk (*P < 0.05 and ** *P* < 0.01 using Students’ *t*-test). (C) Representative images of cell dissociation among wild-type (WT) RMG (I, II) and Snail1 KO1 (III, IV) cells. Before seeding, cells were incubated for 72 h in the absence (I, III) or presence (II, IV) of TGF-beta (10 ng/mL). Photos were taken of whole well (I, III) and of 10 images per sample (II, IV). Scale bar indicates 50 μm. Experiments were repeated twice. (D) Determination of the relative number of fragmented particles, presented as the number of fragmented particles of each type of cells shown in (C) divided by the number of fragmented particles of WT cells incubated in the absence of TGF-beta. Values are expressed as the mean ± s.e.m of ten images per sample. Experiments were repeated twice.

It has been reported that TGF-beta increases cell migration [22]. Indeed, treatment of WT cells with TGF-beta significantly increased migration (Fig 5A-I, II, 5B and S2A Fig-I, II). Although TGF-beta treatment increased migration of Snail1 KO1 cells, the level of migration was significantly lower than that of WT cells (Fig 5A-III, IV, 5B and S2A Fig-III, IV). These data suggest that Snail1 plays an important role in TGF-beta-induced cell migration in RMG cells.

Because TGF-beta treatment reduced the expression of E-cadherin in Snail1 KO1 cells, we assumed that cell–cell adhesion might be reduced by TGF-beta treatment of Snail1 KO1 cells. To test this assumption, we performed a dissociation assay. Treatment with trypsin alone allowed WT and Snail1 KO1 cells to detach in large sheets that were resistant to pipetting-based mechanical dissociation (Fig 5C-I, III and S2B Fig-I, III). Trypsin without EDTA should not inhibit the activity of Ca^2+^-dependent cell adhesion molecules such as E-cadherin. On the other hand, WT and snail KO cells incubated with TGF-beta became detached in small particles upon trypsin treatment. These particles of WT cells were completely dissociated after pipetting (Fig 5C-II, 5D and S2B Fig-II). The particles of Snail1 KO1 cells formed larger aggregates (Fig 5C-IV, 5D and S2B Fig-IV). These data suggest that Snail1 might play an important role in TGF-beta-regulated cell–cell adhesion.

## Discussion

Targeted gene inactivation via homologous recombination has been a powerful method for evaluating the function of genes of interest [23]. However, the use of this technique has been hampered by low inactivation efficiency and the need for time-consuming effort [10]. Targeted gene knockdown by RNAi, a rapid and inexpensive technique, is an alternative to the above-mentioned technology. However, RNAi-mediated knockdown is frequently incomplete and its inhibition is temporary [10]. These restrictions impede the verification of a direct connection between genotype and phenotype. In contrast to these systems, the recently developed CRSIPR/Cas9 system enables the precise editing of specific genomic loci and facilitates the elucidation of target gene function [24]. However, single and double mismatches between the gRNA and DNA sequence are tolerated, leading to readily detected off-target alterations in partially mismatched sites [13]. Thus, we have employed a double-nicking strategy using the Cas9 nickase mutant (Cas9n) with paired guide RNAs to minimize off-target cleavages [14]. In this study, we successfully constructed a Snail1-KO RMG1 cell line using the double-nicking approach. These cells were morphologically different from their parental RMG1 cells, showing a more rounded morphology (Fig 2A-I, II) that appears to be correlated with scarce stress fibers in the cytoplasm and abundant cortical actins (Fig 2A-III, IV).

The cortical actin cytoskeleton is reorganized during EMT. New actin-rich membrane projections called lamelipodia or filopodia appear. Finally, EMT is characterized by increased actin stress fiber formation [1]. Silencing Snail1 in oral squamous cell carcinoma (OSCC) reduced the formation of filopodia and the premature assembly of stress fibers [9]. However, the molecular mechanisms controlling F-actin dynamics during EMT remain to be elucidated [1]. Recently, however, McGrail et al. tried to probe how Snail1-induced EMT produces cytoskeletal changes in epithelial (MCF-7) cells [8]. Contrary to the prevailing theory at the time, they argued that overexpression of Snail1 in MCF7 cells dissolved stress fibers and produced more rounded cells. MCF-7 cells overexpressing Snail1 showed a 3-fold decrease in polymerized F-actin compared with control MCF-7 cells. McGrail et al. assumed that the loss of actin structure was mediated via decreased expression of actin crosslinking proteins, such as filamins A and B. In our study, Snail1 KO1 cells showed an abundance of cortical actins. As the cortical actins consist of F-actin, this result does not contradict theirs. However, Snail1 KO1 cells also showed reduced numbers of stress fibers, did not show the increased expressions of filamin A and B RNA (Data not shown). Although we have not proved it, the upregulation of E-cadherin in Snail1 KO cells might contribute the abundance of cortical actins. Engl et al. recently reported that actin dynamics modulate the immobilization of E-cadherin [25]. E-cadherin forms clusters at the contact rim in an actin-anchoring-dependent manner. We assume that the actin cortex might also be regulated by E-cadherin. Because Snail1 KO cells showed greater expression of E-cadherin than WT cells, knockout of Snail1 may enhance the immobilization of cortical actin at the contact rim. Consistent with our results, Chen et al. reported that E-cadherin-deficient cells showed thicker and more numerous actin stress fibers [26]. Alternatively, increased actin expression might help inhibit the induction of stress fiber formation in Snail1 KO cells. O’Connor reported [27] that cells constitutively expressing active myocardin-related transcription factors (MRTFs, also known as MKL) exhibit marked stress fiber formation with the loss of cortical actin bundles. Translocation of MRTF-A to the nucleus is necessary to induce expression of target genes. Once in the nucleus, MRTF-A regulates the expression of cytoskeletal association proteins [27]. Nobuse reported [28] that an increase in monomeric G-actin leads to the interaction of G-actin with MKL1/MRTF and prevents nuclear translocation of MKL1/MRTF. In our study, Snail1 KO cells showed increased expression of beta-actin. Although we have not assessed the levels of G-actin, increased actin expression might inhibit the nuclear translocation of MKL1/MRTF and inhibit the induction of stress fiber formation in Snail1 KO cells.

Our attempt to obtain a stable Snail1 transfectant probably failed because our Snail1 KO1 cells retained pX335-Cas9-g Snail1 in their genome. This retention may hamper chromosomal integration of a Snail1 cDNA expression unit by the endogenous CRISPR/Cas 9 itself.

We have previously shown that Snail1 enhances cell adhesion to the substrate [6]. Snail1 overexpressing cells exhibit increased expression of integrins alpha 5 and alpha V [6]. Consistent with this data, the Snail1 KO cells showed reduced expression of integrin alpha 5 (Fig 2B and 2C) and cell–substrate adhesion (Fig 3A and 3B). By contrast, Snail1 KO cells did not show reduced adhesion to wells precoated with serum-containing medium. It is possible that the reduction of cell–substrate adhesion might result from reduced ECM deposition in Snail1 KO1 cells. However, fibronectin and vitronectin expression were not reduced in Snail1 KO1 cells, although the secretion of these proteins might be reduced. It is also possible that precoated fibronectin and vitronectin might contribute effective binding to integrin alpha 5. Alternatively, the expression of other extracellular matrix protein might be reduced in Snail1 KO cells. The other issue remaining unclear is the mechanism by which Snail1 KO1 + Snail1 cells showed complete reduction of adhesion to coverslips. This phenomenon occurred in spite of increased expression of fibronectin and vitronectin and comparable expression of integrin alpha 5 in Snail1 KO1 cells. Further analysis is needed.

Shields et al. [29] recently showed that Snail1 expression increases the scattering activity of pancreatic cancer cells. Indeed, ablation of the Snail1 gene leads to a significant reduction in cell scattering. Loss of Snail1 increased cell–cell adhesion and decreased cell dissociation in RMG1 cells. TGF-beta-treated WT and Snail1 KO cells dissociated into small particle upon trypsin treatment and the particles of dissociated Snail1 KO1 cells were larger than those of WT cells (Fig 5C-II, IV and 5D). These results indicated that snail1 plays a role in TGF-beta induced cell dissociation. TGF-beta treatment reduced the expression of E-cadherin in both WT and snail KO cells but the expression of E-cadherin in snail KO cells is still higher than that of WT cells (Fig 4B and 4C). Therefore, the reduction of E-cadherin by TGF–beta would cause cell dissociation and higher expression of E-cadherin might decrease cell dissociation in Snail1 KO1 cells.

Surprisingly, even after treatment with trypsin-EDTA and subsequent washing with PBS (–), it was difficult to dissociate cellular sheets of Snail1 KO1 cells into smaller fragments (Fig 3E-II and 3F). Trypsin-EDTA treatment inhibits the activities of Ca^2+^-dependent cell–cell adhesion molecule such as E-cadherin. Therefore, Snail1 KO cells maintained Ca^2+^-independent cell–cell adhesion. In other word, Snail1 might inhibit Ca^2+^-independent cell–cell adhesion.

Snail1 gene-deficient mice deficient die at the gastrulation stage, at which point the complete EMT process fails [30]. They form an abnormal mesodermal layer that retains epithelial characteristics and E-cadherin expression. Batlle et al. [21] recently demonstrated that Snail1 is required to maintain mesenchymal stem cells in response to TGF-beta signaling [21]. Therefore, we assumed that Snail1 KO cells would fail to complete the EMT process induced by TGF-beta. In reality, Snail1 KO cells showed morphological changes and alterations of gene expression upon TGF-beta treatment (Fig 4). TGF-beta signaling promotes EMT through transcriptional regulation of transcription factors including Slug, Twist, ZEB1 and ZEB2, as well as Snail1 [31]. These factors regulate each other in an elaborate manner [31]. In our study, Snail1 KO cells showed enhanced expression of *ZEB1*, *ZEB2 and TWIST* that was further enhanced by TGF-beta treatment (Fig 4B). Although the mechanism underlying the increased expression of ZEB1and Twist in Snail1 KO cells remains unclear, these factors may have been involved in the induction of morphological changes and alteration of gene expression in TGF-beta-treated Snail1 KO cells. On the contrary, TGF-beta-induced cell migration was significantly reduced in Snail1 KO1 cells. These data indicate that Snail1 play a predominant role in TGF-beta induced cell migration.

Consistent with our results, RNAi-mediated Snail1 KD cells also showed upregulation of epithelial markers, reduced cell migration [9], [32], reducted cell adhesion [33] and reduced cell scattering [20]. However, there are some inconsistencies between the results obtained with Snail1 KO cells and those obtained with snail1 KD cells. For example, ZEB1 expression was reduced in Snail1 KD cells and the upregulation of ZEB1 and TWIST by TGF-beta treatment was blunted in Snail1 KD cells [34]. Indications of TGF-beta-induced EMT, such as morphological change and reduced E-cadherin level, were significantly attenuated in Snail1 KD cells [22]. Although we cannot prove it yet, upregulation of ZEB1 and TWIST in TGF-beta-treated Snail1 KO cells could be a compensatory mechanism. Compared with the generation of stable Snail1 KD cells [22], [34], making Snail1 KO cells took much more time. During the selection process and the confirmation of Snail1 depletion, the cells may have acquired compensation mechanism for the loss of Snail1.

In summary, the precise deletion of Snail1 using the CRISPR/Cas9 system provided clear evidence for the function of Snail1; it is required to maintain actin cytoskeletal structure and cell migration, as well as cell–substrate adhesion, but it suppresses cell–cell adhesion. This study also clarified the redundancy of transcription factors required for the induction of EMT in response to exogenous TGF-beta in RMG1 cells.

## Supporting Information

S1 Fig Loss snail1 altered cytoskeletal organization. Representative images displaying the cell morphology of wild-type (WT) RMG cells (I), Snail1 KO1 cells (II). Cells are visualized by phase-contrast microscopy. Scale bar indicates 50 μm. Representative images of the actin cytoskeletons of WT cells (III) Snail1 KO1 cells (IV) stained with rhodamine X-conjugated phalloidine and visualized using a confocal laser scanning microscope (Zeiss LSM700). Photos of 3 images per sample were taken. Experiments were repeated 5 times. WT cells are rich in stress fibers, whereas Snail1 KO1 cells have scarce stress fibers, but cortical actin on their cell surfaces. Scale bar indicates 20 μm
(TIF)

S2 Fig Loss of snail impeded TGF-beta induced cell migration and cell dissociation. (A) Representative images of wild-type (WT) RMG (I, II) and Snail1 KO1 (III, IV) cells that migrated to the lower surface of the membranes of transwell plates. Before seeding into the transwell plates, cells were incubated for 72 h in the absence (I, III) or presence (II, IV) of TGF-beta (10 ng / mL). Photos were taken of 10 images per sample. Experiments were repeated twice. (B) Representative images of cell dissociation among wild-type (WT) RMG (I, II) and Snail1 KO1 (III, IV) cells. Before seeding, cells were incubated for 72 h in the absence (I, III) or presence (II, IV) of TGF-beta (10 ng/mL). Photos were taken of whole well. Scale bar indicates 50 μm. Experiments were repeated twice.
(TIF)
