# Supplementary figures and images for: CRISPR/Cas9n-Mediated Deletion of the Snail 1Gene (SNAI1) Reveals Its Role in Regulating Cell Morphology, Cell-Cell Interactions, and Gene Expression in Ovarian Cancer (RMG-1) Cells
Source: PLoS One. 2015 Jul 10;10(7):e0132260. doi: 10.1371/journal.pone.0132260 (PMC4498756; doi:10.1371/journal.pone.0132260)

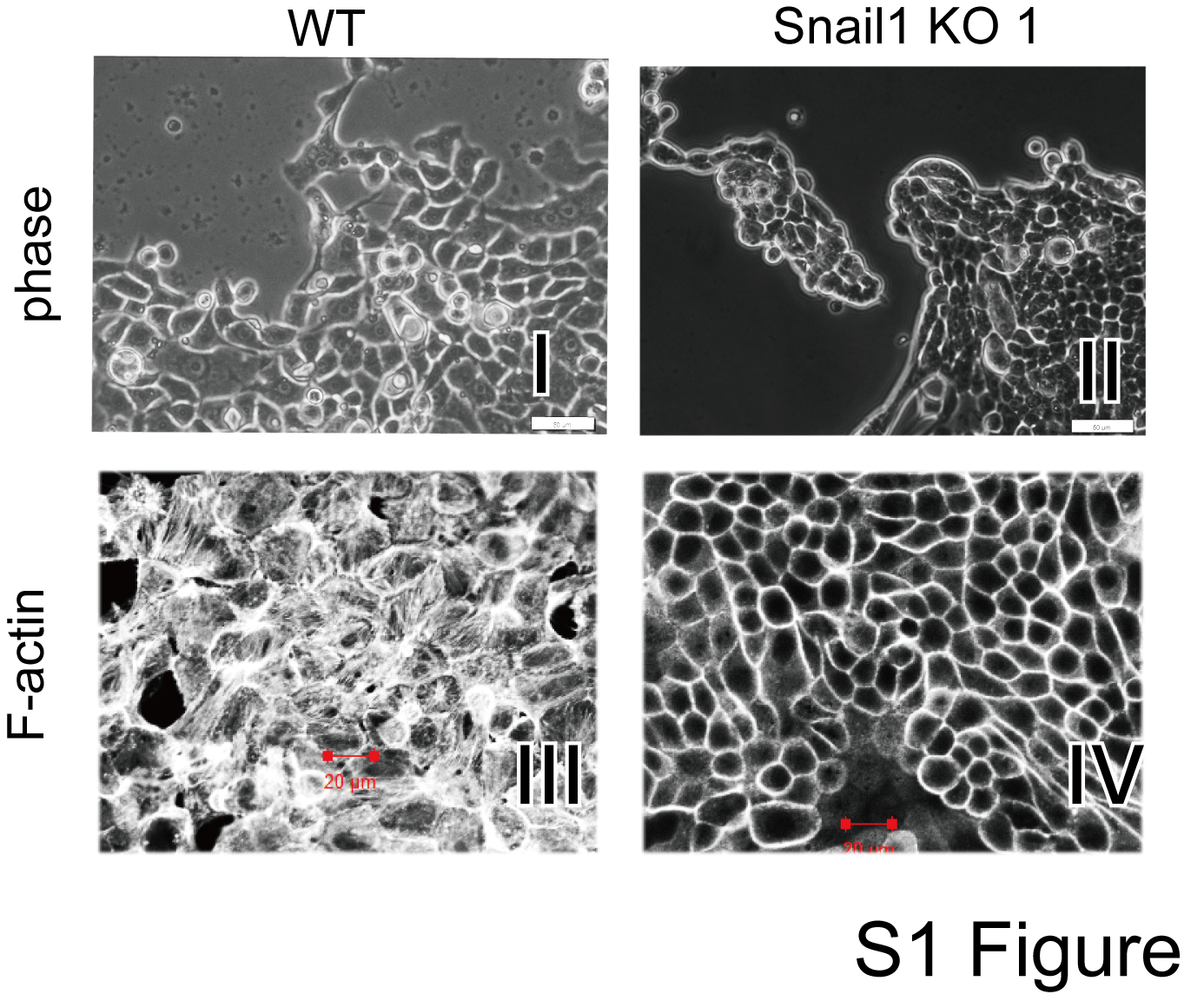

Supplement: S1 Fig — Representative images displaying the cell morphology of wild-type (WT) RMG cells (I), Snail1 KO1 cells (II). Cells are visualized by phase-contrast microscopy. Scale bar indicates 50 μm. Representative images of the actin cytoskeletons of WT cells (III) Snail1 KO1 cells (IV) stained with rhodamine X-conjugated phalloidine and visualized using a confocal laser scanning microscope (Zeiss LSM700). Photos of 3 images per sample were taken. Experiments were repeated 5 times. WT cells are rich in stress fibers, whereas Snail1 KO1 cells have scarce stress fibers, but cortical actin on their cell surfaces. Scale bar indicates 20 μm (TIF) [file pone.0132260.s001.tif]

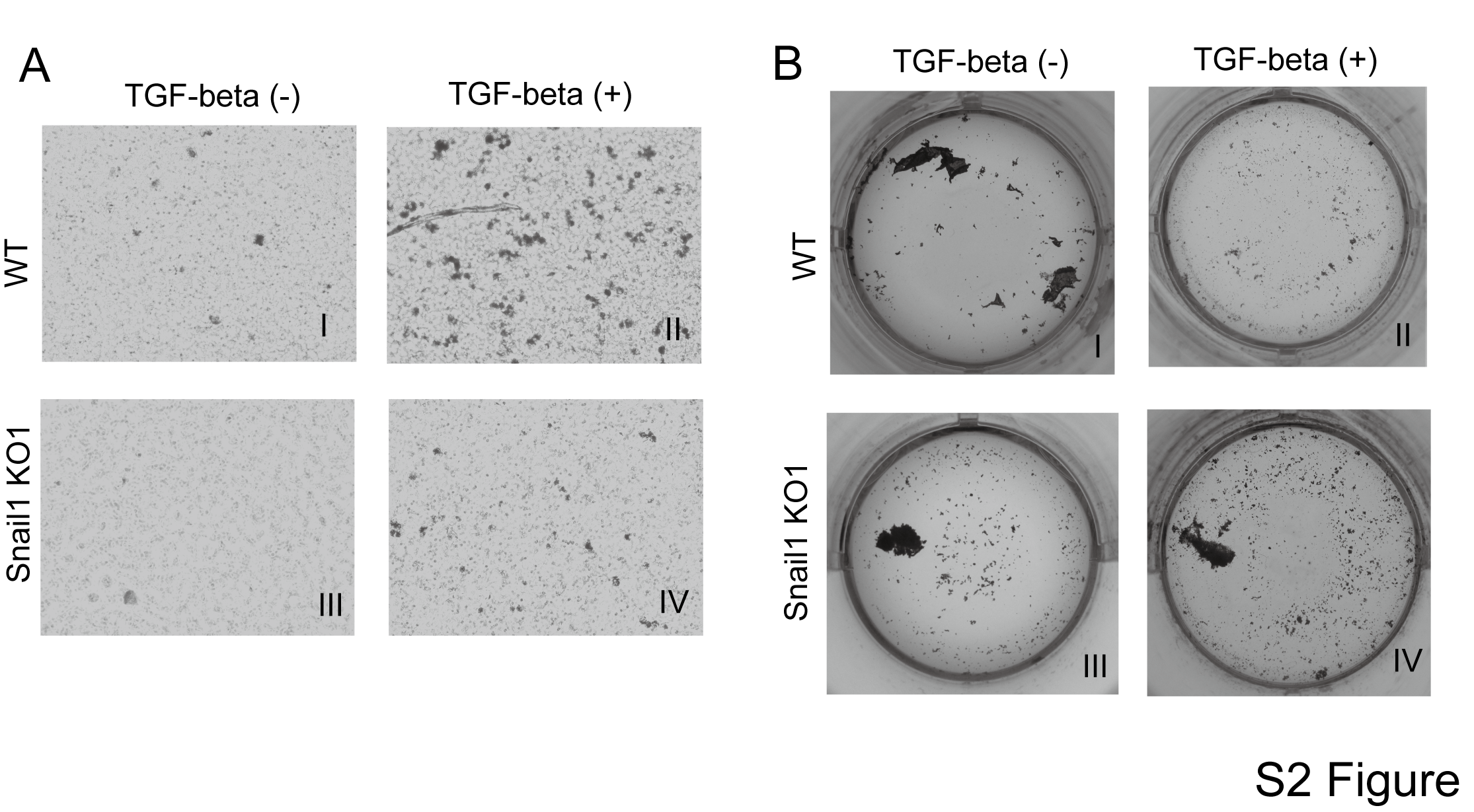

Supplement: S2 Fig — (A) Representative images of wild-type (WT) RMG (I, II) and Snail1 KO1 (III, IV) cells that migrated to the lower surface of the membranes of transwell plates. Before seeding into the transwell plates, cells were incubated for 72 h in the absence (I, III) or presence (II, IV) of TGF-beta (10 ng / mL). Photos were taken of 10 images per sample. Experiments were repeated twice. (B) Representative images of cell dissociation among wild-type (WT) RMG (I, II) and Snail1 KO1 (III, IV) cells. Before seeding, cells were incubated for 72 h in the absence (I, III) or presence (II, IV) of TGF-beta (10 ng/mL). Photos were taken of whole well. Scale bar indicates 50 μm. Experiments were repeated twice. (TIF) [file pone.0132260.s002.tif]
